# Effect of psilocybin on marble burying in ICR mice: role of 5-HT1A receptors and implications for the treatment of obsessive-compulsive disorder

**DOI:** 10.1038/s41398-023-02456-9

**Published:** 2023-05-10

**Authors:** Sandeep Singh, Alexander Botvinnik, Orr Shahar, Gilly Wolf, Corel Yakobi, Michal Saban, Adham Salama, Amit Lotan, Bernard Lerer, Tzuri Lifschytz

**Affiliations:** 1 Biological Psychiatry Laboratory and Hadassah BrainLabs Hadassah Medical Center, Hebrew University Jerusalem, Jerusalem, Israel; 2 Department of Psychology, School of Sciences Achva, Academic College Municipality of Be’er Tuvia, Tuvia, Israel

**Keywords:** Pharmacology, Diseases

## Abstract

Preliminary clinical findings, supported by preclinical studies employing behavioral paradigms such as marble burying, suggest that psilocybin may be effective in treating obsessive-compulsive disorder. However, the receptor mechanisms implicated in the putative anti-obsessional effect are not clear. On this background, we set out to explore (1) the role of serotonin 2A (5-HT2A) and serotonin 1A (5-HT1A) receptors in the effect of psilocybin on marble burying; (2) the effect of staggered versus bolus psilocybin administration and persistence of the effect; (3) the effect of the 5-HT1A partial agonist, buspirone, on marble-burying and the head twitch response (HTR) induced by psilocybin, a rodent correlate of psychedelic effects. Male ICR mice were administered psilocybin 4.4 mg/kg, escitalopram 5 mg/kg, 8-hydroxy-2-(di-n-propylamino) tetralin (8-OH-DPAT) 2 mg/kg, M100907 2 mg/kg, buspirone 5 mg/kg, WAY100635 2 mg/kg or combinations, intraperitoneally, and were tested on the marble burying test. HTR was examined in a magnetometer-based assay. The results show that (1) Psilocybin and escitalopram significantly reduced marble burying. The effect of psilocybin was not attenuated by the 5-HT2A antagonist, M100907. The 5-HT1A agonist, 8-OH-DPAT, reduced marble burying as did the 5-HT1A partial agonist, buspirone. The effect of 8-OH-DPAT was additive to that of psilocybin, but that of buspirone was not. The 5-HT1A antagonist, WAY100635, attenuated the effect of 8-OH-DPAT and buspirone but not the effect of psilocybin. (2) Psilocybin injections over 3.5 h had no effect on marble burying and the effect of bolus injection was not persistent. (3) Co-administration of buspirone with psilocybin blocked its effect on HTR. These data suggest that neither 5-HT2A nor 5-HT1A receptors are pivotally implicated in the effect of psilocybin on marble burying. Co-administration with buspirone may block the psychedelic effects of psilocybin without impeding its anti-obsessional effects.

## Introduction

Obsessive-compulsive disorder (OCD) is a serious, often disabling disorder that affects 100–150 million people with an estimated worldwide prevalence of up to 2% [1]. It is characterized by uncontrollable, recurring thoughts (obsessions) that cause significant distress and by behaviors and actions that the sufferer has the urge to repeat over and over. Available treatments include tricyclic antidepressants such as clomipramine that block serotonin reuptake, specific serotonin reuptake inhibitors (SSRIs), augmentation with second-generation antipsychotic drugs that are 5-HT2A receptor blockers, and exposure and response prevention, a form of cognitive behavioral therapy [2]. At least a third of patients with OCD do not respond to first-line treatments [3]. There is a clear need for therapeutic alternatives.

Research on the clinical application of psychedelics in psychiatry is growing apace after several decades in which their use was legally proscribed [4, 5]. These drugs, particularly the classical tryptaminergic psychedelics, act principally but not only via the 5-HT2A receptor to induce profound changes in perception, cognition, and mood [6, 7]. There is intriguing but limited preliminary evidence that psychedelics, specifically psilocybin, may have a role in the treatment of OCD. This includes a case report on the effectiveness of self-medication with psilocybin-containing magic mushrooms in a patient with long-standing OCD [8]. A preliminary clinical trial of a range of psilocybin doses in nine patients yielded positive results, which were not necessarily related to the induction of psychedelic effects [9]. There are no published reports since the Moreno et al. [9] study but several clinical trials of psilocybin in OCD and related disorders are currently underway: (https://clinicaltrials.gov/ct2/results?cond=Obsessive-Compulsive+Disorder&term=psilocybin&cntry=&state=&city=&dist=, Accessed January 6, 2023).

In the preclinical context, the marble-burying test (MBT) uses the propensity of rodents to dig and cover non-threatening objects placed in their cages as a predictor of anti-obsessional effects [10]. Although criticized as a model of OCD [11], MBT has reasonable predictive validity in terms of identifying drugs that are effective in OCD and potential treatments for the disorder [12]. Studies with MBT have yielded results supportive of the potential therapeutic effect of psychedelics in OCD. Two studies have shown the effect of psilocybin to significantly reduce marble burying in mice [13, 14]. Similar results have been reported for 2,5-dimethoxy-4-iodoamphetamine (DOI) [14] and for the highly selective, brain-penetrant 5-HT2A receptor agonist N-(2-hydroxybenzyl)–2, 5-dimethoxy-4-cyanophenylethylamine (25CN-NBOH) [15].

Psilocin, the active metabolite of psilocybin, binds to 5-HT2A receptors with high affinity [16]. Psychedelic effects of psilocybin in humans are blocked by the 5-HT2A receptor antagonist, ketanserin [17], as is the effect of psilocybin to induce the head twitch response (HTR) in mice [18, 19], a characteristic rodent behavior that is correlated with psychedelic effects in humans [20]. The anti-marble-burying effect of psilocybin does not appear to be mediated by 5-HT2A receptors since it is not blocked by prior administration of the highly selective 5-HT2A antagonist, M100907 (volinanserin) [14]. It is noteworthy, however, that M100907 does block the effect of DOI to reduce marble burying [15]. Odland et al. [14] also showed that the effect of psilocybin on marble burying was not blocked by the 5-HT2C receptor antagonist, SB242084, suggesting that this receptor is also not implicated in the anti-marble-burying effect of psilocybin.

In the current study, we sought to re-examine the role of 5-HT2A receptors in the effect of psilocybin on marble burying and to explore the role of 5-HT1A receptors by studying 5-HT1A agonist, partial agonist, and antagonist effects in the MBT in conjunction with psilocybin. We also examined the effect of staggered psilocybin administration as compared to bolus injection and the persistence of the anti-marble-burying effect after 7 days.

## Materials and methods

### Ethical statement

Experiments were conducted in accordance with AAALAC guidelines and were approved by the Authority for Biological and Biomedical Models Hebrew University of Jerusalem, Israel, Animal Care and Use Committee (Project No. MD-21-16663-4 approved on 22/8/2021 and Project No. MD-21-16754-4 approved on 12/10/2021). All efforts were made to minimize animal suffering and the number of animals used.

### Reagents and chemicals

Chemically synthesized psilocybin (PSIL) was kindly supplied by USONA Institute, Madison, Wisconsin, USA, and was determined by Ultra Performance Liquid Chromatography (UPLC) to contain 98.75% psilocybin. Escitalopram (ESC), 8-hydroxy-dipropylamino-tetralin hydrobromide (8-OH-DPAT) (DPAT), volinanserin (M100907), and buspirone (BUSP) were of highest purity grade and were purchased from Sigma Aldrich Israel Ltd, Israel. WAY100635 (WAY) was obtained from Biotest (Tocris Bioscience), Israel. 8-hydroxy-2-(di-n-propylamino) tetralin (8-OH-DPAT) and M100907 were dissolved in vehicle (0.9% saline) (VEH) containing 5% dimethyl sulfoxide (DMSO). Psilocybin, escitalopram, WAY100635, and buspirone were dissolved in a 0.9% saline vehicle.

### Animals and experimental design

The study was carried out on male ICR mice (30.0 ± 2.0 g). The animals were housed in a controlled environment (25 ± 2 °C, relative humidity 55 ± 15%) with a 12-h light/dark cycle. All mice were fed with a normal laboratory diet of nutrient-rich pellets ad libitum. The experimental mice were allowed to acclimatize prior to starting the experiment. Separate sets of mice were used for the MBT and head twitch response (HTR) experiments. The sample size was estimated on the basis of prior studies. Formal randomization and blinding were not applied.

Drugs were administered by intraperitoneal (i.p.) injection in a standard injection volume of 300 µl 30 min before the MBT. Mice were divided into the following treatment groups: *Vehicle (VEH):* 0.9% saline; *Psilocybin (PSIL)*: 4.4 mg/kg dissolved in the vehicle. (This dose was chosen because, in a preliminary experiment, we found that PSIL 1.5 mg/kg (as used by Matsushima et al. [13] in male ICR mice, did not significantly affect marble burying); *Escitalopram (ESC)*: 5 mg/kg dissolved in vehicle; *8-OH-DPAT (DPAT)*: 2 mg/kg dissolved in vehicle containing 5% DMSO; *M100907*: 2 mg/kg dissolved in vehicle containing 5% DMSO; *Buspirone (BUSP)*: 5 mg/kg dissolved in vehicle; *WAY100635 (WAY)*: 2 mg/kg dissolved in vehicle; *M100907*+*PSIL*; *DPAT*+*PSIL*; *WAY*+*PSIL*; *BUSP*+*PSIL*.

### Marble-burying test (MBT)

MBT (see Supplementary Video) was performed in transparent cages containing ~4.5 cm fine sawdust, as described by Odland et al. [14]. Twenty glass marbles were placed equidistant from each other in a 5 × 4 pattern. The experiment was done under dim light in a quiet room to reduce the influence of anxiety on behavior. The mice were left in the cage with the marbles for a 30-min period, after which the test was terminated by removing the mice. A marble was considered buried when two-thirds or more of its size was covered with burying substrate, and the number of buried marbles was counted after 30 min. All mice underwent a pretest without any injection, and the number of marbles buried was counted. Only mice that buried at least 15 marbles were selected to perform the test after drug administration. Eighty percent of pretested mice fulfilled this criterion and were used in the definitive experiment, which took place at least a week following the pretest.

### Open field test (OFT)

The OFT was performed immediately after the MBT to evaluate the effects of the drug treatments on locomotor activity. The apparatus consisted of a square wooden arena (50 × 50 × 40 cm) with white walls and a black floor. Mice were placed individually in the center of the open field and allowed to freely explore the apparatus for 30 min. A camera was used to monitor movement. The total distance traveled (centimeters) was measured by the Ethovision XT-12 Video Tracking System (Noldus Information Technology BV). After each test, the arena was cleaned with a 70% alcohol solution.

### Head twitch response

Head twitch response (HTR) was measured over 20 min by means of a magnetometer apparatus as described by Revenga et al. [21] and Shahar et al. [22]. Briefly, small neodymium magnets (N50, 3 mm diameter × 1 mm height, 50 mg) were attached to the outer ears of mice. After a 5- to 7-day recovery period, the ear-tagged animals were placed inside a magnetometer apparatus (supplied by Mario de la Fuente Revenga, PhD., of Virginia Commonwealth University) immediately after injection of vehicle or psilocybin 4.4 mg/kg. The output was amplified (Pyle PP444 phono amplifier) and recorded at 1000 Hz using a NI USB-6001 (National Instruments, US) data acquisition system. Recordings were performed using a MATLAB driver (MathWorks, US, R2021a version, along with the NI myDAQ support package) with the corresponding National Instruments support package for further processing. A custom MATLAB script was used to record the processed signal, which was presented as graphs showing the change in current as peaks (mAh). A custom graphical user interface created in our laboratory was used to further process the recording into an Excel spreadsheet.

### Statistical analysis

The experimental data in all figures are expressed as the mean ± standard error of the mean (SEM). (Versions of the figures that show individual datapoints are provided in the Supplementary Data section). To determine intergroup differences, one-, two-, and three-way analysis of variance (ANOVA) were used as indicated. Bonferroni’s or Tukey’s multiple comparison tests were used to analyze post-hoc comparisons. *p* < 0.05 (two-tailed) was the criterion for significance. GraphPad Prism, version 9.3.1 software, was used for all statistical analyses.

## Results

### Marble-burying test

**Fig. 1:**
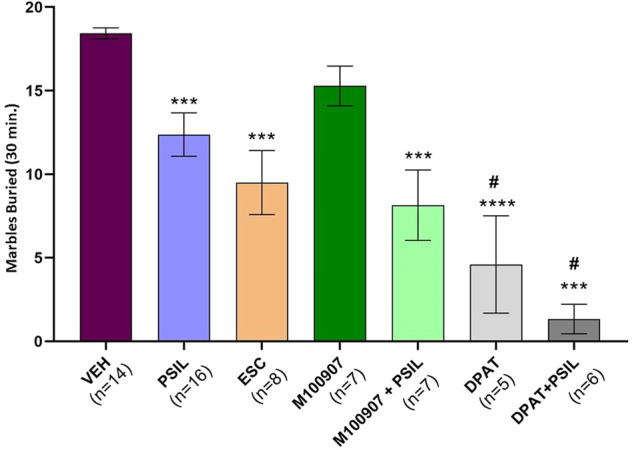
Psilocybin reduces marble burying - Role of 5-HT2A and 5-HT1A receptors. Effect of psilocybin 4.4 mg/kg, escitalopram 5 mg/kg, M100907 2 mg/kg, and M100907 2 mg/kg + psilocybin 4.4 mg/kg, 8-OH-DPAT 2 mg/kg and 8-OH-DPAT 2 mg/kg + psilocybin 4.4 mg/kg on total marbles buried over 30 min. One-way ANOVA: F_6,56_ = 15.33 *p* < 0.0001. **p < 0.01 vs. VEH, #*p* < 0.01 vs. PSIL, *n* = 8–16 (Bonferroni’s multiple comparisons test).

Mice administered psilocybin buried 32.84% fewer marbles over 30 min than vehicle-treated mice (*p* = 0.001, Fig. 1). The effect of psilocybin was not statistically different from the effect exerted by a positive control, the SSRI escitalopram (48.43% reduction in marble burying relative to vehicle; *p* < 0.0001, Fig. 1). Using a two-way-ANOVA design, we examined the effects of acute treatment with psilocybin and pretreatment with the 5-HT2A antagonist, M100907 (volinanserin) (Fig. 1). A strong main effect of psilocybin was noted (F_1,40_ = 24.8, *p* < 0.0001). We also observed a main effect of M100907 (F_1,40_ = 7.7, *p* = 0.008, Fig. 1). However, there was no interaction between psilocybin and M100907 and the post-hoc comparison of M100907 and vehicle was not significant (*p* > 0.10) while the post-hoc comparison of M100907+psilocybin and vehicle was (*p* < 0.0001). These findings indicate that the mechanism whereby psilocybin reduces marble-burying behavior is likely independent of 5-HT2A receptor signaling.

**Fig. 2:**
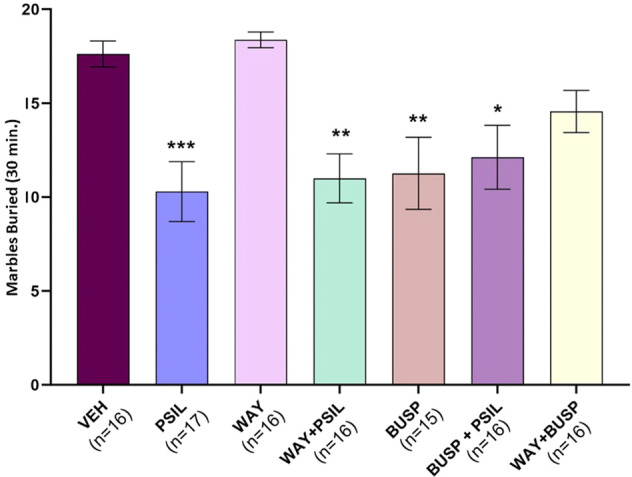
Psilocybin, buspirone and WAY100635 - effect on marble burying. Effect of psilocybin 4.4 mg/kg, WAY100635 2 mg/kg, WAY100635 2 mg/kg + psilocybin 4.4 mg/kg, buspirone 5 mg/kg, buspirone 5 mg/kg+ psilocybin 4.4 mg/kg and WAY100635 2 mg/kg + buspirone 5 mg/kg on total marbles buried over 30 min. One-way ANOVA: F_6,105_ = 6.045, *p* < 0.0001. **p < 0.01 vs. VEH, #*p* < 0.01 vs. PSIL, *n* = 15–17 (Bonferroni’s multiple comparisons test).

Could stimulation of 5-HT1A receptors underlie psilocybin’s effect on marble burying? Psilocybin and the 5-HT1A agonist, 8-OH-DPAT, both exerted significant main effects to reduce marble burying (F_1,37_ = 10.4, *p* = 0.0026 and F_1,37_ = 74.3, *p* < 0.0001, respectively, Fig. 1). However, psilocybin did not interact with 8-OH-DPAT (F_1,37_ = 0.9, *p* = 0.34 for psilocybin-DPAT interaction term) indicating that 5-HT1A stimulation was unlikely to account for psilocybin’s effect in the marble-burying paradigm. Moreover, the combined effect of psilocybin and 8-OH-DPAT was significantly greater than that of vehicle or psilocybin alone (*p* < 0.0001 and *p* < 0.0001, respectively), implying an additive effect of the two drugs. To consolidate this observation, we tested the inhibitory effect of pretreatment with the 5-HT1A receptor antagonist, WAY100635, before psilocybin administration. Using a two-way-ANOVA design, we noted a strong main effect of psilocybin (F_1,61_ = 42.47, *p* < 0.0001, Fig. 2), while the main effect of WAY100635 and the psilocybin-WAY100635 interaction were both not significant (F_1,61_ = 0.4, *p* = 0.521 and F_1,61_ = 0.0003, *p* = 0.985, respectively, Fig. 2). Post-hoc testing confirmed that pretreatment with WAY100635 does not block the effect of psilocybin to reduce marble burying (*p* > 0.10, Fig. 2). As high doses of both psilocybin and WAY100635 were used, we assume maximal receptor occupancy of both substances so that any 5-HT1A-mediated effect of psilocybin would have been blocked by WAY100635, hence concluding that psilocybin effect on MB is not 5-HT1A-mediated.

**Fig. 3:**
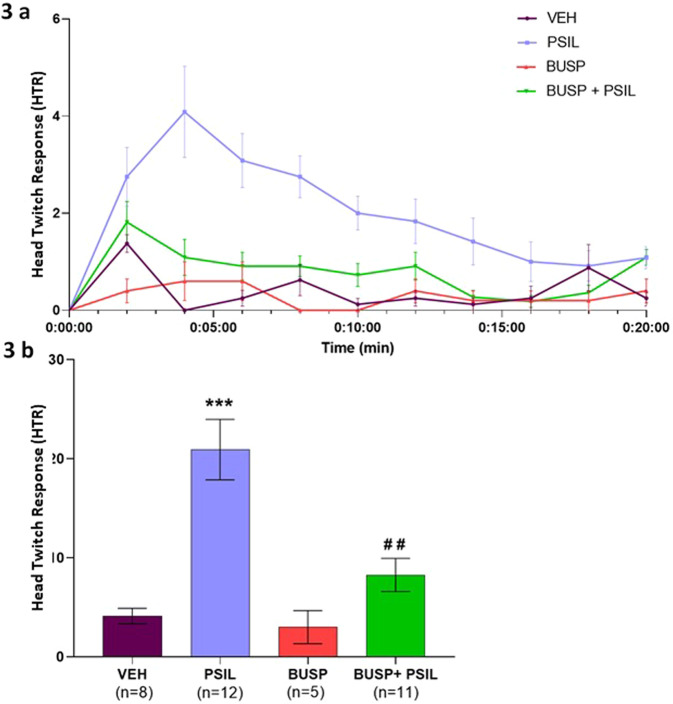
Psilocybin, buspirone and the head twitch response. **a** Effect of psilocybin 4.4 mg/kg, buspirone 5 mg/kg and psilocybin 4.4 mg/kg + buspirone 5 mg/kg on HTR over a 20-min measurement period. Three-way ANOVA: Time F_9,288_ = 5.001, *p* = 0.0032; Time x psilocybin F_9,288_ = 3.224, *p* = 0.001; Time × psilocybin × buspirone F_9,288_ = 2.687, *p* = 0.0072 (within subject effects). psilocybin F_1,32_ = 19.22, *p* = 0.0001; buspirone F_1,32_ = 7.483, *p* = 0.0101; psilocybin × buspirone F_1,32_ = 5.237, *p* = 0.0289 (between-subject effects). **b** Total HTR over 20 min. F_3,32_ = 12.87, *p* < 0.0001; ***p < 0.001 vs. vehicle, ##*p* = 0.0009 buspirone + psilocybin vs. psilocybin, *n* = 6–12 (Tukey’s multiple comparisons test).

Buspirone is a 5-HT1A receptor partial agonist and a weak dopamine D2 receptor antagonist [23, 24]. Unlike 8-OH-DPAT, we found an interaction of buspirone with psilocybin (F_1,75_ = 5.805, *p* = 0.018, Fig. 2). Thus, although psilocybin and buspirone alone both significantly reduced marble burying compared to vehicle (F_1,75_ = 6.53, *p* = 0.0; F_1,75_ = 68.53, *p* = 0.005; respectively, Fig. 2), their co-administration did not yield a further reduction in marble burying. We also tested the effect of pretreatment with WAY100635 on the reduction in marble burying induced by buspirone (buspirone F_1,59_ = 19.45, *p* < 0.0001, WAY100635 F_1,59_ = 3.07, *p* = 0.08, WAY100635 X buspirone F_1,59_ = 1.2, *p* = 0.274, Fig. 3b). Contrasting with the lack of effect of WAY100635 on the psilocybin-induced reduction in marble burying (Fig. 2), we found a significant effect of WAY100635 to attenuate the effect of buspirone on marble burying (buspirone vs. vehicle, *p* < 0.001; WAY100635+buspirone vs. vehicle, *p* > 0.10) although the comparison of WAY100635+buspirone versus buspirone was not significant.

### Open field test

Mice were placed in an open field on completion of the MBT and were monitored for 30 min using the Ethovision XT-12 Video Tracking System (Noldus Information Technology BV). As shown in Supplementary Fig. 1a, there was no significant difference in distance traveled between vehicle-treated mice and those administered psilocybin or buspirone. Similarly, there was no difference in time spent by the mice in the center of the open field (center duration) (Supplementary Fig. 1b) or in the periphery of the open field (periphery duration) (Supplementary Fig. 1c) under treatment with psilocybin or buspirone compared to treatment with vehicle.

### Persistence of effect

A key question not addressed in studies thus far is whether the effect of psilocybin and other psychedelic compounds on marble burying is transient or persistent. We examined marble burying in a subset of mice treated with vehicle or psilocybin 7 days following the initial MBT. No significant effect of psilocybin was observed (vehicle 18.6 ± 1.6, *n* = 5; psilocybin 17.75 ± 2.21, *n* = 4; *p* = 0.53) (Supplementary Fig. 2).

### Requirement for bolus administration

We further examined whether the effect of psilocybin on marble burying requires a bolus injection of the full dose of the drug (at 4.4 mg/kg) or whether the same effect can be achieved by administering the same quantity of drug in a staggered fashion over a period of 3.0 h, i.e., i.p. injections of 1.1 mg/kg every 60 min (four in total) with the MBT performed 30 min after the last injection. When administered in this fashion, no significant effect of psilocybin on marble-burying was observed (vehicle 19 ± 0.89, *n* = 6; psilocybin 19 ± 1.32, *n* = 9; *p* > 0.10) (Supplementary Fig. 3).

### Evaluation of HTR

To determine whether the dose of psilocybin that inhibited marble burying in this study would induce a significant increase in the number of head twitches observed in ICR mice as compared to vehicle, HTR was measured in a magnetometer device as described above. We further examined the effect of co-administration of buspirone 5 mg/kg with psilocybin 4.4 mg/kg to determine whether co-administration of buspirone would attenuate the HTR-enhancing effect of psilocybin. Figure 3 shows the time course of the effect of psilocybin and buspirone on HTR. Three-way ANOVA with repeated measures showed a significant within-subjects effect of time (F_9,288_ = 5.001, *p* < 0.0032), reflecting the changes in HTR rate during the course of the test; a time by psilocybin interaction (F_9,288_ = 3.224, *p* = 0.001), reflecting differential, psilocybin-dependent changes in the HTR rate during the course of the test and a triple, time by psilocybin by buspirone interaction (F_9,288_ = 2.687, *p* = 0.0072), reflecting differential psilocybin- and buspirone-dependent, changes in the HTR rate over time. Significant between-subject effects of psilocybin (F_1,32_ = 19.22, *p* = 0.0001) and buspirone were observed (F_1,32_ = 7.483, *p* = 0.0101) and a significant psilocybin by buspirone interaction (F_1,32_ = 5.237, *p* = 0.0289) indicating effects over time. When evaluating the total number of HTRs during the 20-min measurement period (Fig. 4b), there were significant effects of psilocybin (F_1,32_ = 19.22, *p* = 0.0001) and buspirone (F_1,32_ = 7.483, *p* = 0.0101) and a significant psilocybin × buspirone interaction (F_1,32_ = 5.237, *p* = 0.0289). In post-hoc tests, there was a significant effect of psilocybin to increase HTR compared to vehicle (*p* < 0.001), while buspirone and buspirone + psilocybin effects were significantly lower than the effect of psilocybin (*p* = 0.0002 and *p* = 0.0009, respectively).

## Discussion

The results of our study support a significant effect of psilocybin to reduce the number of marbles buried in the MBT by male ICR mice when the drug is administered 30 min before the test, as previously reported by Matsushima et al. [13] and by Odland et al. [14]. Matsushima et al. [13] studied male ICR mice and observed a significant effect of psilocybin at a dose of 1.5 mg/kg. Odland et al. [14] studied female NMRI mice and observed a significant effect of psilocybin at 1.0 mg/kg. In our study, 1.5 mg/kg was not sufficient to obtain a significant effect on male ICR mice, and we used a dose of 4.4 mg/kg psilocybin for our experiments. This dose was chosen on the basis of prior dose-response studies on the effect of psilocybin on HTR [22]. The reason for this difference between our study and that of Matsushima et al. [13] is not clear.

As reported by Odland et al. [14], we found that the 5-HT2A antagonist, M100907 (volinanserin), did not block the effect of psilocybin on marble burying. While Odland et al. [14] used 0.1 mg/kg of M100907, we administered a considerably higher dose (2.0 mg/kg) in order to be certain that dosage is not the reason for non-blockade of the effect of psilocybin on marble burying by M100907. It should be taken into account, however, that this high dose of M100907 could induce non-specific effects [23]. In contrast, the anti-marble-burying effect of the 5-HT2A agonist, DOI, could be blocked by M100907 [14]. While the reason for this discrepancy is not clear, our data and those of Odland et al. [14]. are consistent with psilocybin exerting its effects through mechanisms other than 5-HT2A signaling.

Our findings regarding the role of 5-HT1A receptors in the effect of psilocybin on marble burying are intriguing. As previously reported by others [25, 26], the prototypical 5-HT1A agonist, 8-OH-DPAT, significantly reduced marble burying in our study. Combined administration of 8-OH-DPAT and psilocybin exerted an additive anti-marble-burying effect. There was no interaction between 8-OH-DPAT and psilocybin, consistent with a differential mode of action exerted by these two compounds. Moreover, pretreatment with the 5-HT1A antagonist, WAY100635, which has been shown to block the effect of 8-OH-DPAT on marble burying [27] and was shown to have this effect for the 5-HT1A partial agonist, buspirone, in our study, did not attenuate the effect of psilocybin. The dose of WAY100635 that we used (2 mg/kg) was sufficient since it has been shown by Harasawa et al. [27] that 1 mg/kg of WAY100635 was sufficient to block the marble-burying effect of 30 mg/kg of fluvoxamine [27]. Taken together, these two findings suggest that the effect of psilocybin to reduce marble burying is not mediated by the 5-HT1A receptor but by a different, as yet unelucidated mechanism.

While the 5-HT1A partial agonist, buspirone, significantly reduced marble burying in our study, its effect was not additive to the effect of psilocybin as was the case for 8-OH-DPAT and psilocybin. We deduce from the additive effects of 8-OH-DPAT and psilocybin on MB behavior that the effect of psilocybin on MB is not mediated via 5-HT1A receptors; hence the effects of the two drugs are non-competitive. However, if the effect of buspirone on MB behavior is mediated via 5-HT1A receptors, whereas the effect of psilocybin on MB is mediated via another mechanism that is possibly antagonized by buspirone, the effects of the two drugs might be competitive rather than additive. In a study that tested the effects of 8-OH-DPAT and buspirone on changes in the levels of striatal dopamine, serotonin, and their metabolites, 8-OH-DPAT was demonstrated to decrease striatal 5-HIAA/5-HT ratio but had no effect on striatal HVA/DA ratio, whereas buspirone increased striatal HVA/DA ratio but had no effect on the 5-HIAA/5-HT ratio [28]. Also, although the two compounds are known for their inhibitory effect on burying behavior, the effect of buspirone is reported to be mediated via adrenocorticoid secretion, whereas the effect of 8-OH-DPAT is not glucocorticoid-dependent [29]. Interestingly, whereas the behavioral effects of 8-OH-DPAT are mostly anxiolytic, buspirone was demonstrated to evoke an anxiogenic effect and reduced locomotion. The 5-HT1A antagonist WAY100635 did not block the anxiogenic or motor effects of buspirone, suggesting that, unlike the anxiolytic effect, these effects are not 5-HT1A-mediated [30]. Taken together, these data suggest that whereas some of the effects of 8-OH-DPAT and buspirone are 5-HT1A-mediated, other effects rely on other pathways, which might account for the differential effects of these two compounds.

It should further be noted that antipsychotic drugs, which have been demonstrated to have an inhibitory effect on marble burying, exert their effect via several targets, including D2 and 5TH2A receptors (see, for example, Egashira et al. [30]). Moreover, WAY100635 had no effect on the reduction of MB induced by aripiprazole [26], although aripiprazole is a known partial agonist of 5-HT1A receptors. Although our data suggest that buspirone exerts its effect on marble burying via interaction with 5-HT1A receptors, the interaction of buspirone with other targets, such as D2 receptors and alpha-2 adrenergic receptors, might be relevant in the context of OCD.

As noted, we have demonstrated that buspirone inhibited HTR induced by psilocybin, an observation that is consistent with the finding of Pokorny et al. (2016) [31] that buspirone attenuated the psychedelic effects of psilocybin in healthy volunteers (although these effects are usually considered as 5-HT2A-mediated [31]. We have similarly shown that HTR is inhibited by the 5-HT1A receptor agonist, 8-OH-DPAT [22]. The mechanism whereby 5-HT1A agonism inhibits HTR is not presently known and requires further study. In this regard, it is noteworthy that 5-HT1A and 5-HT2A receptors are co-expressed and have been shown to interact negatively [32]. Other mechanisms, such as dopaminergic and adrenergic effects, should be considered, although these are rendered less likely by the strong effect of the high-affinity 5-HT1A agonist, 8-OH-DPAT, to reduce HTR [22]. The clinical implication of this finding is that co-treatment with psilocybin and buspirone could potentially permit the anti-obsessional effects of psilocybin while blocking its psychedelic effects. Further exploration of this interaction is an important topic for further study that has high relevance for the treatment of OCD.

As previously demonstrated by Odland et al. [14], the effect of psilocybin and buspirone to reduce marble burying in our study was not a consequence of reduced motor activity. In our measurements of open field activity, there was no difference in the effect of psilocybin and buspirone compared to vehicle. Our activity measurements were not performed during the MBT, 30–60 min after the psilocybin injection but after the MBT, 60–90 min after psilocybin administration. Nevertheless, it is noteworthy that the activity measurements performed by Odland et al. (14) were during the MBT, and no difference in the effect of psilocybin compared to the vehicle was observed. A further concern is that continued head twitching may have resulted in reduced marble burying by the mice that received psilocybin. However, we have recently shown that head twitching is virtually absent 30 min after administration of psilocybin 4.4 mg/kg [22], and we evaluated marble burying 30–60 min after psilocybin. Moreover, we did not observe head twitching during the time mice were being evaluated for marble burying.

We did not find an effect of psilocybin on marble burying that extended beyond that observed after 30 min. No effect was observed when a subset of mice was retested after 7 days. We did not test the effect after 24 h, which should also be done to provide further insights regarding the time course of the anti-obsessional effect of psilocybin. Also, we found that a bolus injection of psilocybin was needed to achieve an effect on marble burying and there was no effect of psilocybin on the MBT when the same dose was spaced over 4 h rather than being administered at once. Implications for the anti-obsessional effect of psilocybin in humans await studies in which the longer-term effects of psilocybin administration on OCD are examined, and the dose required is clarified. In the study by Moreno et al. (2006), clinical observations were not performed beyond 24 h following oral administration of psilocybin [9]. However, doses of psilocybin that did not induce prominent psychedelic effects were found to have anti-obsessional effects, while in our study, a bolus of drug sufficient to induce prominent HTR in mice was required, as demonstrated by our HTR data.

In considering the limitations of our study, it should be noted that since the experiments were performed in male mice only, the results are not generalizable to females. Furthermore, viewing the MBT as a screening test with reasonable predictive validity for therapeutic effects in OCD [12], our findings should be tested in OCD models that have greater face and construct validity [11].

Although the present study confirmed the inhibitory effect of psilocybin on marble burying that was recently reported by Odland et al. [14] and previously by Matsushima et al. [13], and that this effect is not mediated via 5-HT2A receptors and most likely not via 5-HT1A receptors, the exact mechanism through which psilocybin exerts this effect is still yet to be determined. There is evidence that psilocin, the active metabolite of psilocybin, has a high affinity for the serotonin transporter and also for TAAR1 receptors [33]. Both these targets could be explored as potential mechanisms for an anti-obsessional effect of psilocybin that is not mediated by the 5-HT2A receptor. Furthermore, the complex interaction between psilocybin and buspirone suggests that psilocybin might affect dopaminergic and/or noradrenergic pathways. Such effects can be tested by measuring levels of 5-HT, dopamine, and norepinephrine following psilocybin administration in the frontal cortex as well as in subcortical areas such as the striatum. In this context, it is noteworthy that Wojtas et al. [34] found that psilocybin (2 and 10 mg/kg) increased dopamine, serotonin, glutamate, and GABA extracellular levels in the frontal cortex, while psilocybin also increased GABA in the reticular nucleus of the thalamus. C-fos expression following MBT in psilocybin-treated mice may shed further light on the brain mechanisms that underlie the effect of psilocybin on MB. It would be interesting to visualize C-fos also in mice treated with buspirone in addition to psilocybin in order to test the interaction of the two compounds.

In considering the translational relevance of our findings, dosage issues should be considered. Based on the allometric scaling for dose conversion from mice to humans proposed by Nair and Jacob [35], our psilocybin dose of 4.4 mg/kg in mice would be equivalent to 0.35 mg/kg in humans (25 mg in a 70 kg person) which is a frequently used psychedelic dose in humans. Similarly, our buspirone dose in mice (5 mg/kg) would translate to 0.40 mg/kg in humans (28 mg in a 70 kg person), which is well within the clinical dose range of buspirone.

In conclusion, the results of our study confirm the previously reported effect of psilocybin to reduce marble burying in mice, confirm that this effect is not blocked by a 5-HT2A receptor antagonist, and suggest that the effect is not mediated by 5-HT1A receptors since the effects of psilocybin and 8-OH-DPAT are additive and the effect of psilocybin is not blocked by the 5-HT1A antagonist, WAY100635. The results further show that co-treatment with the 5-HT1A partial agonist, buspirone, blocks the effects of psilocybin on HTR, a rodent correlate of psychedelic effects, while not impeding its effect on marble burying. Further studies are indicated to identify the receptor mechanisms of psilocybin in reducing marble burying in mice with implications for the mechanism of the putative anti-obsessional effect of psilocybin in humans.

## Supplementary information

Singh et al, Supplemental Material

Supplemental video
