# Supplementary material for: Effect of psilocybin on marble burying in ICR mice: role of 5-HT1A receptors and implications for the treatment of obsessive-compulsive disorder
Source: Transl Psychiatry. 2023 May 10;13:164. doi: 10.1038/s41398-023-02456-9 (PMC10172379; doi:10.1038/s41398-023-02456-9)
Supplement: Supplementary file 1 — Singh et al, Supplemental Material [file 41398_2023_2456_MOESM1_ESM.pdf]

Singh et al. Supplemental Material

Supplemental Fig. 1

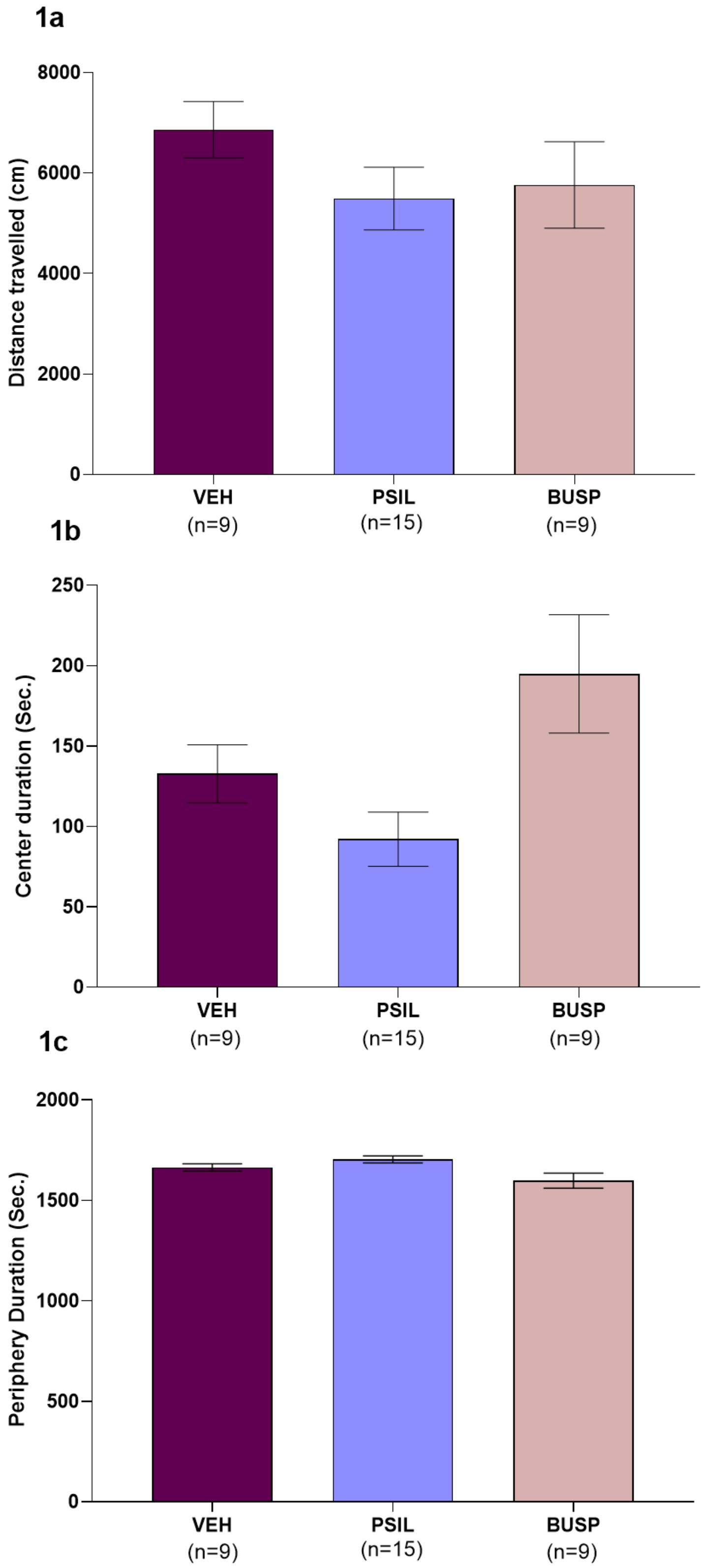

Supplemental Fig. 1: **1a**: Effect of psilocybin 4.4 mg/kg and buspirone 5 mg/kg on distance travelled in the open field over 30 minutes. One way ANOVA: $F_{2, 30}$ = 1.044; p=0.3645 . p N.S. vs. VEH.

1b: Effect of psilocybin 4.4 mg/kg and buspirone 5 mg/kg on time spent in the center of the open field over 30 minutes. One way ANOVA: $F_{2, 30}$ = 4.934; p=0.0140. p N.S. vs. VEH. **1c**: Effect of psilocybin 4.4 mg/kg and buspirone 5 mg/kg on time spent in the periphery of the open field over 10 minutes. One way ANOVA: $F_{2, 30}$ = 5.003; p = 0.0133. p N.S vs VEH, n= 9-15 (Tukey's multiple comparisons test).

Supplemental Figure 2

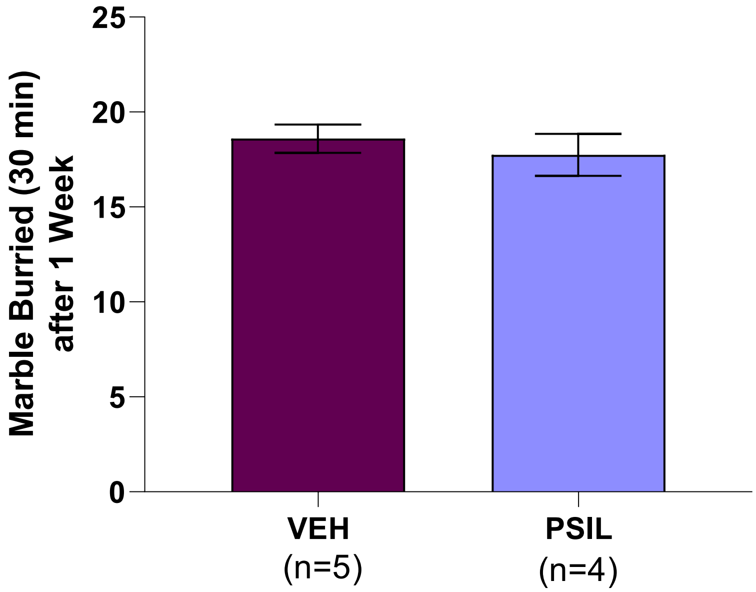

Supplemental Fig. 2: Effect of psilocybin 4.4 mg/kg after 1 week on total marbles buried over 30 minutes. No significant effect of psilocybin was observed (vehicle 18.6 ± 1.6, n=5; psilocybin 17.75± 2.21 n=4; p =0.53).

Supplemental Figure 3

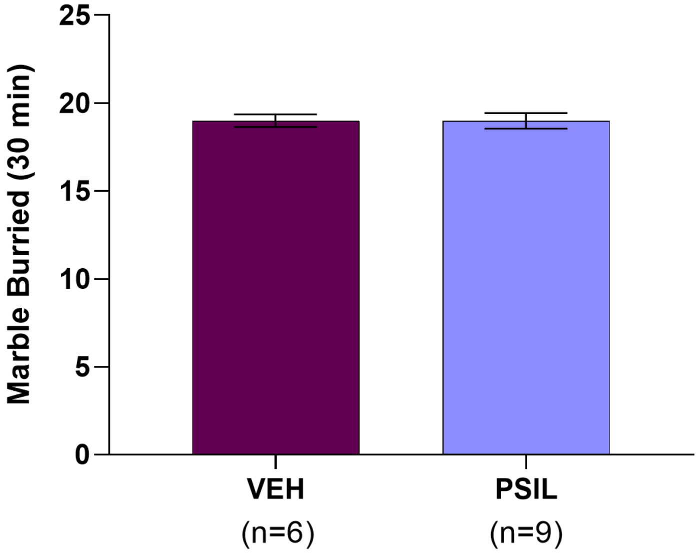

Supplemental Fig. 3: Effect of psilocybin 4.4 mg/kg drug in staggered fashion over a period of 3.30 hours *i.e.* i.p. injections of 1.1 mg/kg every 60 minutes; MBT performed 30 minutes after the last injection. No significant effect of psilocybin was observed (vehicle 19±0.89 n=6; psilocybin 19±1.32 n=9; p>0.10) (n=6-9).

Alternative versions of Figs 1, 2 and 3 showing individual data points

Fig. 1

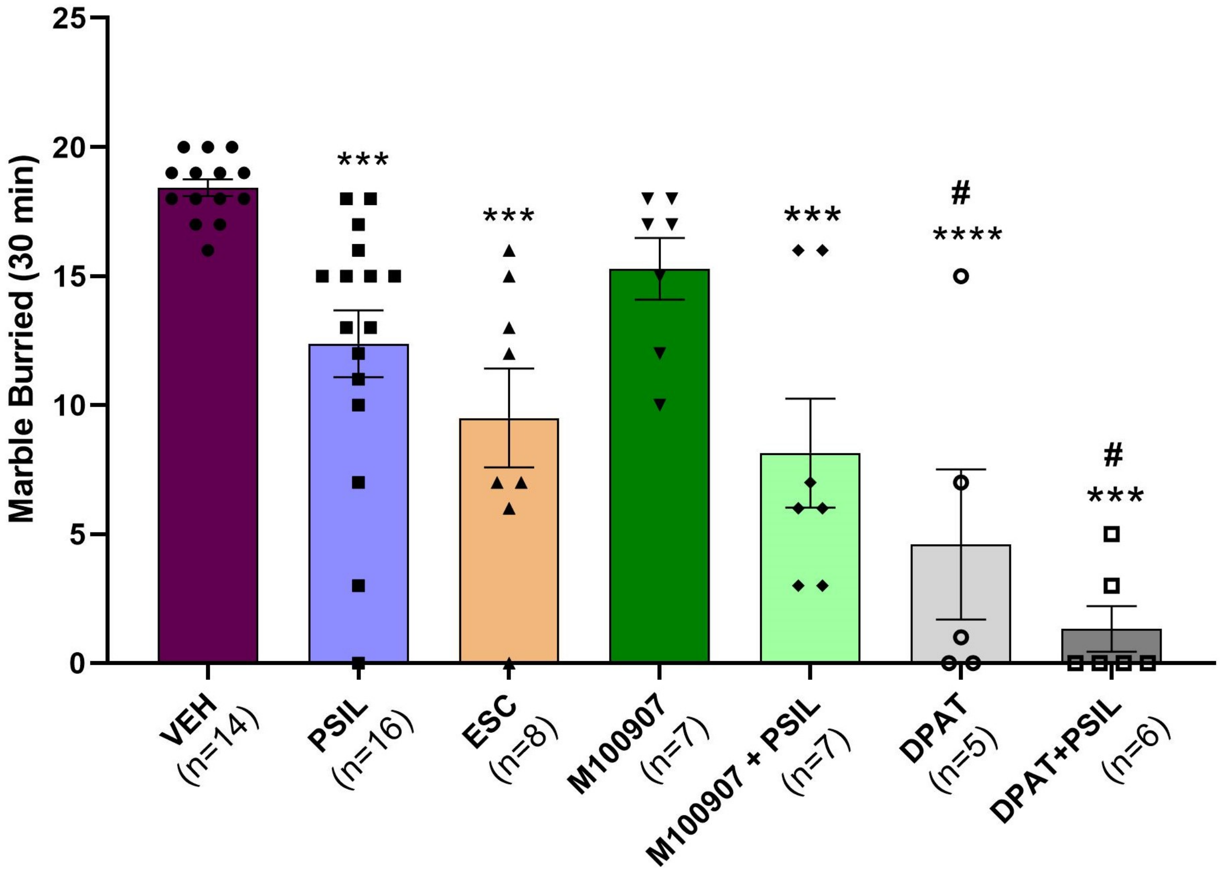

Figure 1: Effect of psilocybin 4.4 mg/kg, escitalopram 5 mg/kg, M100907 2 mg/kg and M100907 2 mg/kg + psilocybin 4.4 mg/kg, 8-OH-DPAT 2mg/kg and 8-OH-DPAT 2 mg/kg + psilocybin 4.4 mg/kg on total marbles buried over 30 minutes. One way ANOVA: $F_{6, 56} = 15.33$ $p<0.0001$. **p<0.01 vs. VEH, # p<0.01 vs. PSIL n=8-16 (Bonferroni's multiple comparisons test).

Fig. 2

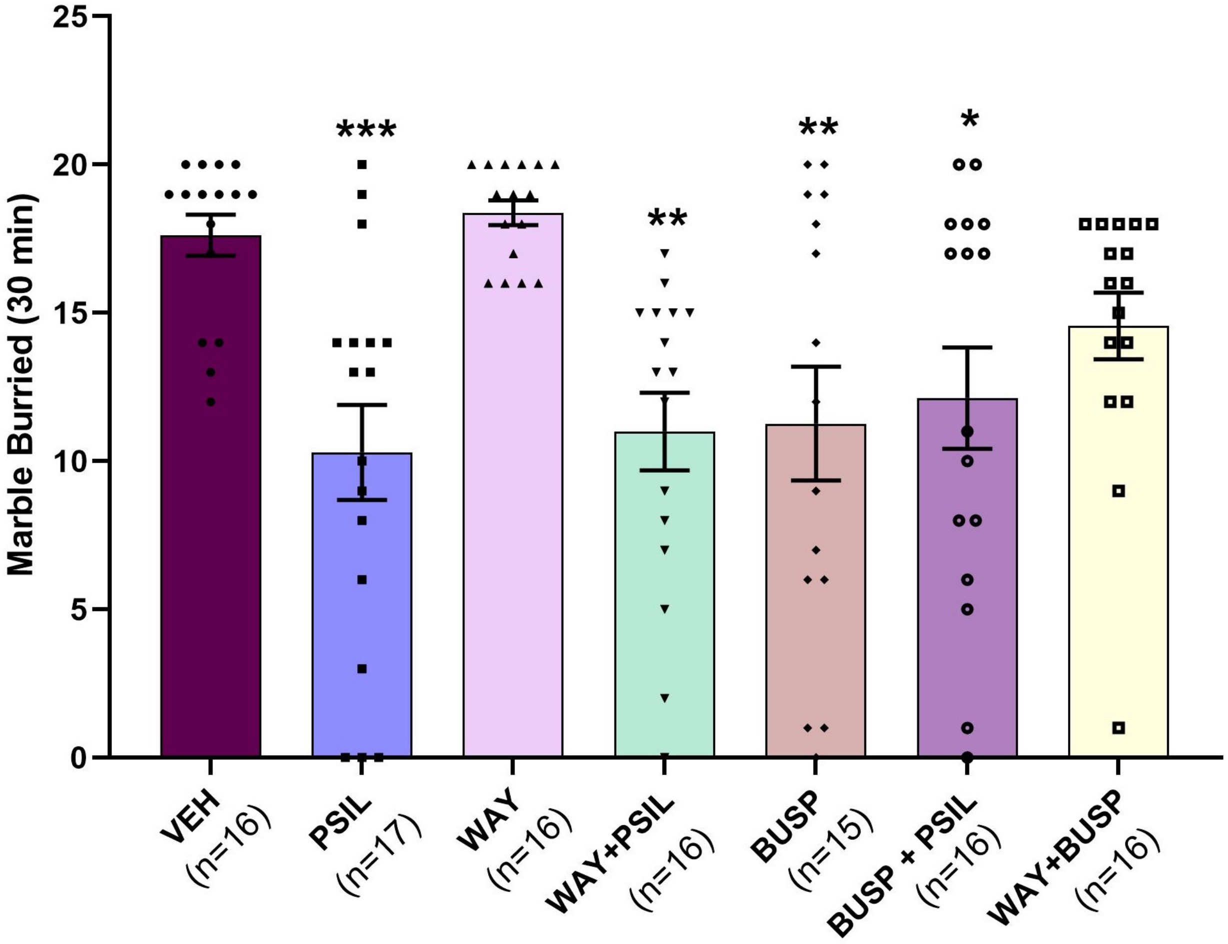

Figure 2: Effect of psilocybin 4.4 mg/kg, WAY100635 2 mg/kg , WAY100635 2 mg/kg + psilocybin 4.4 mg/kg, buspirone 5 mg/kg , buspirone 5 mg/kg+ psilocybin 4.4 mg/kg and WAY100635 2 mg/kg + buspirone 5 mg/kg on total marbles buried over 30 minutes. One way ANOVA: $F_{6, 105} = 6.045$, $p<0.0001$. **p<0.01 vs. VEH, # p<0.01 vs. PSIL n=15-17 (Bonferroni's multiple comparisons test).

Fig. 3

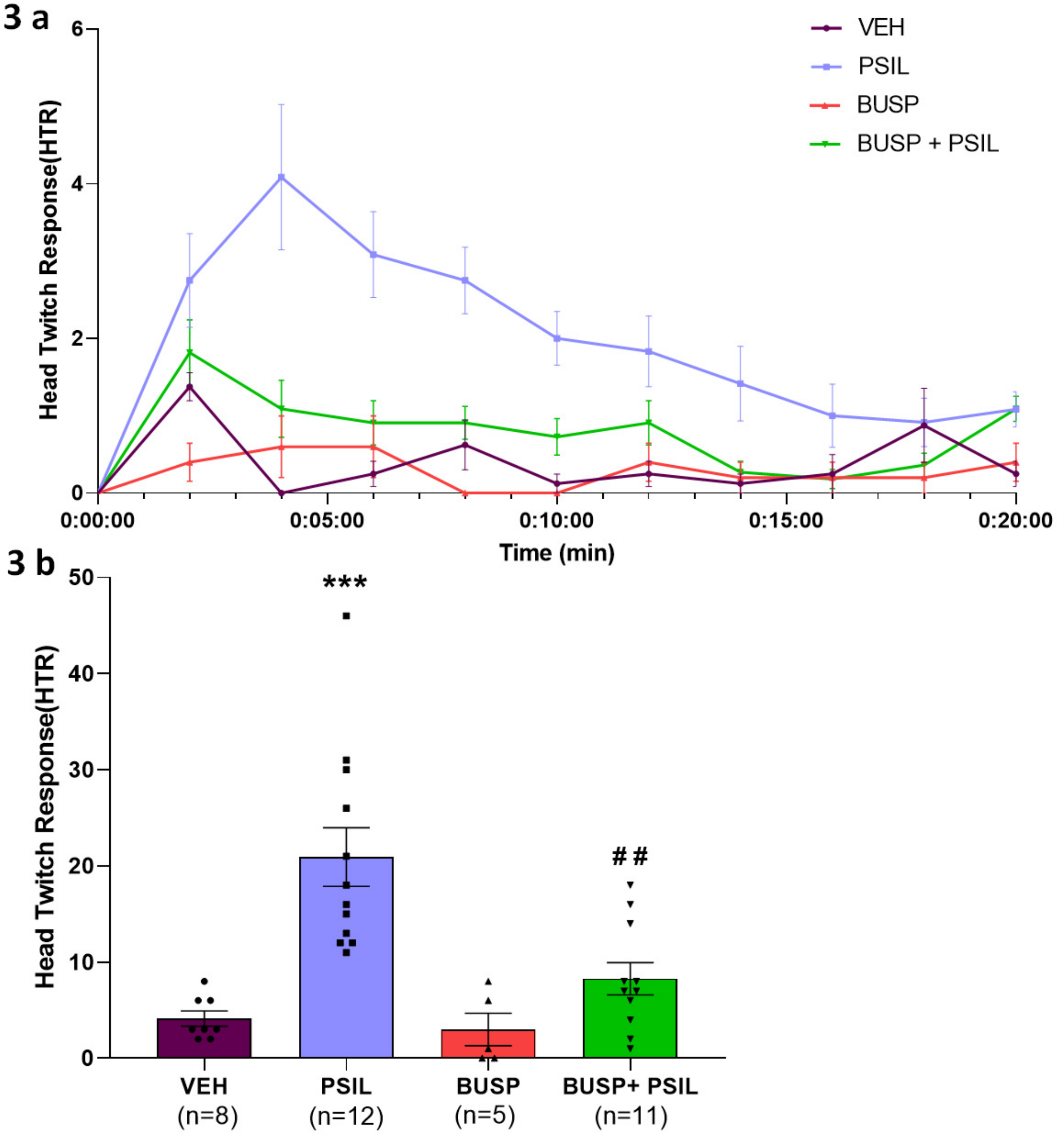

Figure 3: 3a: Effect of psilocybin 4.4 mg/kg, buspirone 5 mg/kg and psilocybin 4.4 mg/kg + buspirone 5 mg/kg on HTR over a 20-minute measurement period. Three-way ANOVA: Time $F_{9,288} = 5.001$, $p = 0.0032$; Time x psilocybin $F_{9, 288} = 3.224$, $p = 0.001$; Time x psilocybin x buspirone $F_{9, 288} = 2.687$, $p=0.0072$ (within subject effects). psilocybin $F_{1, 32} = 19.22$, $p=0.0001$;

buspirone $F_{1, 32}$ = 7.483, p=0.0101; psilocybin x buspirone $F_{1, 32}$ = 5.237, p=0.0289 (between subject effects). **3b:** Total HTR over 20 minutes. $F_{3,32}$ = 12.87, p <0.0001; ***p<0.001 vs. vehicle, ## p=0.0009 buspirone + psilocybin vs. psilocybin, n=6-12 (Tukey's multiple comparisons test).

SUPPLEMENTAL VIDEO

This video shows ICR mice engaged in marble-burying. This behavior serves as the basis for the Marble-burying test. Mice did not receive any pretreatment before being placed in the test cage which contained twenty marbles equidistant from each other in a 5 × 4 pattern. The experiment was done under dim light in a quiet room to reduce the influence of anxiety on behavior. The mice were left in the cage with the marbles for a 30-min period after which the test was terminated by removing the mice. Number of buried marbles was counted after 10, 20 and 30 minutes.

https://drive.google.com/file/d/1n5oKtI4ZyewVwn1suDFXxifdbwjO4Wtt/view?usp=sharing

(Filmed by Dr Alexander Botvinnik, Biological Psychiatry Laboratory and Hadassah BrainLabs, Hadassah Medical Center, Hebrew University, Jerusalem, Israel)
